# Effects of a gluten-free hydrolyzed diet in dogs with paroxysmal dyskinesia: a prospective cohort study

**DOI:** 10.1093/jvimsj/aalag255

**Published:** 2026-10-28

**Authors:** Samuel Okonji, Francesca Del Baldo, Elisa Garuti, Carla G Vecchiato, Alessandro Di Carlo, Gaia Costantini, Alberto Cauduro, Andrea Di Paola, Cristian Falzone, Antonella Gallucci, Ilaria Tartari, Massimo Baroni, Federica Poli, Ezio Bianchi, Valentina Buffagni, Alessia Valentini, Teresa Gagliardo, Gualtiero Gandini

**Affiliations:** Department of Veterinary Medical Sciences, University of Bologna, Bologna, Italy; Veterinaria Corlazzoli, Rome, Italy; Department of Veterinary Medical Sciences, University of Bologna, Bologna, Italy; Department of Veterinary Medical Sciences, University of Bologna, Bologna, Italy; Department of Veterinary Medical Sciences, University of Bologna, Bologna, Italy; Department of Veterinary Medical Sciences, University of Bologna, Bologna, Italy; Department of Veterinary Medical Sciences, University of Bologna, Bologna, Italy; Neurovet, Legnano, Milan, Italy; Neurovet, Legnano, Milan, Italy; Clinica Veterinaria Pedrani, Diagnostica Piccoli Animali Srl, Zugliano, Italy; Veterinary Neurological Center `La Fenice,' Selargius, Italy; Veterinary Neurological Center `La Fenice,' Selargius, Italy; Valdinievole Veterinary Clinic, Monsummano Terme, Italy; Valdinievole Veterinary Clinic, Monsummano Terme, Italy; Department of Veterinary Medical Sciences, University of Parma, Parma, Italy; Department of Veterinary Medical Sciences, University of Parma, Parma, Italy; AniCura Clinica Veterinaria Arcella, Padova, Italy; PalermoVet Veterinary Diagnostic Center, Palermo, Italy; Department of Veterinary Medical Sciences, University of Bologna, Bologna, Italy

**Keywords:** antibodies, canine, dystonia, involuntary movement, movement disorder, seizure

## Abstract

**Background:**

Paroxysmal dyskinesia (PD) is an episodic movement disorder in dogs. Gluten-sensitive etiology has been suggested, but prospective studies in different breeds are lacking.

**Hypothesis/Objectives:**

Investigate whether a gluten-free hydrolyzed diet (GFHD) decreases PD episode frequency and gastrointestinal (GI) signs, and evaluate associations with serum anti-gliadin peptide immunoglobulin G (AGA IgG) and anti-transglutaminase-2 immunoglobulin A (TG-2 IgA) titers.

**Animals:**

Twenty-three client-owned dogs with PD.

**Methods:**

Prospective cohort study with 3 time points: baseline (T0), 90 days (T1), and 180 days (T2). Dogs were fed a GFHD. Episode frequency, GI signs and AGA IgG and TG-2 IgA titers were assessed at each time point. Response to GFHD was classified as excellent (episode disappearance), good (≥50% decrease), fairly good (<50% decrease), or poor (no change or increase).

**Results:**

Median number of episodes decreased from 6 (range, 2-60) at T0 to 2 (range, 0-10) at T2 (*P* = .0003). At T2, 17/23 dogs were responders (5 excellent, 12 good). Gastrointestinal signs were present in 15/23 dogs at T0 and in 6/23 dogs at T2, with significant decreases in vomiting and diarrhea (*P* = .01). No significant correlation was found between episode frequency and AGA IgG or TG-2 IgA titers at any time point (*P* > .1). Antibody results did not significantly change between T0 and T2.

**Conclusions and clinical importance:**

The GFHD was associated with a significant decrease in PD frequency and GI signs in most dogs. The AGA IgG and TG-2 IgA titers were not predictive of dietary response, suggesting limited value as biomarkers.

## Introduction

Paroxysmal dyskinesias (PDs) are a heterogeneous group of self-limiting, involuntary movement disorders that recur episodically and last from a few seconds to several hours.^1^ Episodes are painless and typically do not include autonomic signs, altered consciousness, or abnormal postictal phase behavior.^1^^,^^2^ Video footage of the episode might be helpful in distinguishing PDs from epileptic seizures, one of the main differential diagnoses for PD.^1^ Between the episodes, neurologic examination is normal. Paroxysmal dyskinesia is diagnosed based on medical history, clinical evaluation, and visualization of episodes via video recording.^3^

Classification systems divide PDs into 4 categories based on etiology: genetic (proven and suspected), reactive (dietary, drug-induced, toxic, and metabolic), structural (neoplasia, inflammatory, and other structural causes), and unknown.^3^

In recent years, paroxysmal gluten-sensitive dyskinesia (PGSD), first described in Border Terriers (BTs), has prompted particular interest.^4–8^ In BTs, PGSD was accompanied by gastrointestinal (GI) signs in 24% of cases, including vomiting, diarrhea, and borborygmi.^7^ The relationship between PGSD and GI disorders remains unclear and has not been documented in veterinary medicine.

Reliable serum antibody biomarkers (anti-gliadin peptide immunoglobulin G [AGA IgG] and transglutaminase-2 immunoglobulin A [TG-2 IgA]) have been used to diagnose gluten sensitivity in BTs, as previously described in the diagnosis of celiac disease in human beings.^9^ These antibodies were found to be increased in a study on BTs with canine epileptoid cramping syndrome, a type of PD, compared with a population of dogs with idiopathic epilepsy, other diseases and a healthy control group.^7^

Except for one prospective case series of 6 BTs,^6^ literature on PDs in dogs and clinical responses to gluten-free diets is limited to retrospective investigations with small study populations and lack of standardized diagnostic and therapeutic approaches and inadequate follow-up.^10–14^

In human medicine, gluten-related disorders present with a variety of clinical signs, ranging from GI to neurologic manifestations. Among the latter, PD has increasingly been reported in association with celiac disease, suggesting a possible link between the 2 conditions.^15–17^

The purpose of our prospective study was to evaluate the response to a gluten-free hydrolyzed diet (GFHD) in dogs of all breeds with a clinical diagnosis of PD. Specifically, the primary objective of our study was to verify the effect of a GFHD on the frequency of PD episodes. In addition, we aimed to evaluate changes in AGA IgG and TG-2 IgA titers and, if present at the time of the inclusion, changes in GI signs after introducing the GFHD.

## Materials and methods

### Study design and case selection

Our prospective multicenter cohort study enrolled dogs presented to 8 referral neurology centers between June 2023 and August 2024. The study was approved by the University of Bologna Ethics Committee (Prot ID 133080/2023), and informed owner consent was obtained.

Dogs were eligible if they had experienced ≥ 2 episodes of suspected PD within the preceding 6 months, diagnosis had been confirmed by review of video recordings by an European College of Veterinary Neurology (ECVN) diplomate or resident, interictal neurologic examination was normal, and the dogs had not previously received a gluten-free diet or treatment with antiepileptic or serotonergic medications, carbonic anhydrase inhibitors, or corticosteroid drugs. Brain magnetic resonance imaging (MRI) had to be normal or show only incidental findings unrelated to PD. Dogs without video documentation or receiving treatment for GI disease were excluded.

### Study protocol

Dogs were evaluated before dietary intervention (T0) and after 90 (T1) and 180 (T2) days of an exclusive commercial soy-based GFHD. Baseline assessment included signalment, body condition score (9-point scale),^18^ age at PD onset, episode characteristics, GI signs, physical and neurologic examination, brain MRI, abdominal ultrasonography, CBC, serum biochemistry, serum electrolyte, folate and cobalamin concentrations, fecal parasitological examination, and serum anti-gliadin peptide immunoglobulin G (AGA IgG) and transglutaminase-2 IgA (TG-2 IgA) measurements. The study design is shown in Supplementary Material S1.

Suspected chronic gastroenteropathy was defined as persistent GI signs with supportive ultrasonographic or folate and cobalamin abnormalities or both. Owners maintained standardized diaries recording PD episodes and dietary compliance. Follow-up evaluations included physical and neurologic examination, PD occurrence, GI signs, diet acceptance, and repeat antibody measurements.

### Outcome measures

Dietary efficacy was assessed by comparing PD episode frequency during the 6 months before diet initiation with the T0-T1, T1-T2, and T0-T2 intervals. Response was classified as excellent (no episodes), good (≥50% decrease), fairly good (<50% decrease), or poor (unchanged or increased frequency). Dogs with excellent or good responses were classified as responders. Antibody titers were categorized as negative (<0.6), equivocal (0.6-0.8), or positive (>0.8).

### Statistical analysis

Data distribution was assessed using the Shapiro–Wilk test. Longitudinal changes in antibody titers and PD episode frequency were analyzed using Friedman tests, with Wilcoxon signed-rank tests for paired comparisons. Associations between antibody titers and PD frequency were evaluated using Spearman correlation, and changes in GI signs using McNemar’s test. Statistical significance was set at *P* < .05. Additional methodological details are provided in the Appendix.

## Results

### Animals

A total of 32 dogs were enrolled in the study. Nine dogs were excluded for the following reasons: 5 because of owner noncompliance with the study protocol, 3 because of inadequate acceptance of the diet, and 1 because of introduction of an antiepileptic drug (levetiracetam) throughout the study period. A total of 23 dogs completed the study (Figure 1). Table 1 summarizes the demographic characteristics of the dog population.

**Figure 1:**
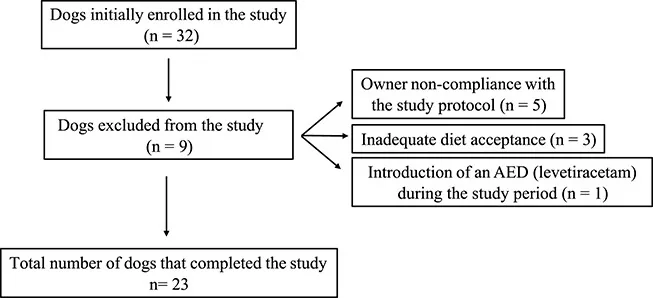
Flowchart of case selection. *n* = number of dogs.

**Table 1:** Demographic characteristics of dogs enrolled in the study (*n* = 23).

**Characteristic**	**Value**
**Breed distribution**	***n* (%)**
- Mixed breeds	6 (26.1%)
- Toy Poodle	4 (17.4%)
- Miniature Pinscher	3 (13.0%)
- Maltese	2 (8.7%)
- Maltipoo	1 (4.3%)
- Maremma Hound	1 (4.3%)
- Boston Terrier	1 (4.3%)
- Cocker Spaniel	1 (4.3%)
- Labrador Retriever	1 (4.3%)
- Scottish Shepherd	1 (4.3%)
- Greyhound	1 (4.3%)
- French Bulldog	1 (4.3%)
**Sex, *n* (%)**	
- Female	10 (43.5%)
- Spayed	9
- Entire	1
- Male	13 (56.5%)
- Entire	10
- Castrated	3
**Body weight, kg**	Median: 7.5 (range: 2.2-41.0)
**Age at T0, years**	Median: 5.0 (range: 2.3-9.7)
**Age at first PD episode, years**	Median: 3.1 (range: 1.6-8.2)
**Comorbidities, *n* (% of total)**	6 (26.1%)
- Chronic gastroenteropathy	3
- Dermatopathy	3

Abbreviation: PD, paroxysmal dyskinesia.

### Occurrence of PD episodes

The median number of PD episodes in the 6 months preceding the introduction of the GFHD was 6 (range, 2-60; Table 2). The median number of PD episodes per dog per month was 1.5 (range, 0.33-10).

Episodes lasted < 1 min in 2 dogs (8.7%), 1-5 min in 10 dogs (43.5%), 5-10 min in 8 dogs (34.8%), 10-30 min in 2 dogs (8.7%), and > 30 min in 1 dog (4.3%). Episodes mainly occurred after excitement or stress in 3 dogs (13.0%), and after meals in 2 dogs (8.7%). No trigger event was identified in 18 dogs (78.3%).

**Table 2:** Characteristics of PD episodes in the dog’s population.

**Variable**	**No. of dogs (%)**	**Details**
**Median number of PD episodes in the 6 months prior to gluten-free diet**	23	6 (range 2-60)
**Identified triggers**		
- Excitement or stress	3 (13.0%)	
- Eating	2 (8.7%)	
- No identifiable trigger	18 (78.3%)	
**Pre-episode phase**		
- Absent	20 (87.0%)	
- Present (owner-reported predictive behavior)	3 (13.0%)	Restlessness (1), mild sialorrhea with nausea (1), attention-seeking behavior (1)
**Clinical signs during the episode**		
**Dystonia**	21 (91.3%)	Abnormal body posture with sustained, involuntary muscle contractions
- All 4 limbs involved	11	Generalized postural abnormality and/or inability to stand
- Forelimbs only	3	
- Hindlimbs only	2	
- Right forelimb only	2	
- Left forelimb only	2	
- Left forelimb and hindlimb	1	
Cervical dystonia	11 (47.8%)	
**Tremors**	17 (73.9%)	Generalized (15), restricted to the head (2)
**Ability to ambulate during the PD episode**		
- Maintained	7 (30.4%)	
- Impaired: o Unable to stand ▪ Sternal recumbency ▪ Lateral recumbency	16 (69.6%) 13 7 6	13 unable to maintain quadrupedal stance
**Level of consciousness**	23 (100%)	Normal
**Autonomic signs**	3 (13.0%)	Vomiting and defecation (1), mild hypersalivation (2) associated with dystonic masticatory muscle contraction
**Post-episode recovery**		
- Immediate return to normal	19 (82.6%)	
- Transient residual signs	4 (17.4%)	Panting, proprioceptive ataxia, mild mydriasis, restlessness
Vomiting after episode	5 (21.7%)	

Abbreviation: PD, paroxysmal dyskinesia.

The PD episodes were analyzed based on videos recorded by the owners. According to the owners, the recorded video episodes could be considered somewhat representative of how PD episodes were manifested in their dogs. The most frequent patterns were dystonia and tremors. Supplementary Material S2 shows video footage of different PD episodes in dogs from our study population. The clinical features of PD episodes observed in the study population are summarized in Table 2.

### Neurologic evaluation and MRI findings at T0

The neurologic examination was normal in all dogs. Magnetic resonance imaging of the brain was normal in 18 dogs (78.3%). Alterations not specifically related to PD were found in 5 dogs (21.7%). Three dogs had mild ventriculomegaly of the lateral ventricles, one dog had a Chiari-like malformation (CLM), and another dog had a fluid collection in the right tympanic bulla.

### Gastrointestinal assessment at T0

Gastrointestinal signs reported by the owner included sialorrhea, nausea, vomiting, diarrhea, borborygmi, and indirect signs of possible abdominal pain (eg, kyphosis, tense abdomen). At T0, 10 dogs (43.5%) had more than one GI sign, 5 (21.7%) had one sign, and 8 (34.8%) had no obvious GI signs. Vomiting was reported in 12 dogs, diarrhea in 10, and borborygmi in 4. Sialorrhea, nausea, and signs consistent with abdominal discomfort were each noted in 2 dogs. Of the 15 dogs (65.2%) with GI signs, 2 (13.3%) had both AGA IgG and TG-2 IgA positive results (median, 1.27 and 1.19, respectively), 5 (33.3%) had an AGA IgG or TG-2 IgA positive result (median, 0.94), 5 (33.3%) had both AGA IgG and TG-2 IgA negative results (median, 0.33 and 0.44, respectively) and one (6.7%) had at least AGA IgG or TG-2 IgA equivocal result (0.63).

Among the 8 dogs (34.8%) without GI signs, 2 (25.0%) had both AGA IgG and TG-2 IgA positive results (median, 2.12 and 1.33, respectively), 4 (50.0%) had AGA IgG or TG-2 IgA positive result (median, 1.54), and 2 (25.0%) had at least one AGA IgG or TG-2 IgA questionable result (median, 0.64). Physical examination was unremarkable in all dogs. Body condition score (BCS) was distributed as follows: a score of 4/9 was observed in 4 dogs (17.4%), 5/9 in 12 dogs (52.2%), 6/9 in 5 dogs (21.7%), and 7/9 in 2 dogs (8.7%).

Fecal parasitological examination was negative in all dogs. Two dogs (8.7%) had increased alanine amino transferase activity, with a median of 194.5 U/L (reference range, 15-65 U/L), whereas 2 dogs (8.7%) had a slight increase in γ-glutamyl transferase activity, with a median of 5.9 U/L (reference range, 0-5 U/L). One (4.4%) dog had increased aspartate aminotransferase activity (123 U/L; reference range, 15-52 U/L). Serum folate concentrations were decreased (median, 2.8 μg/L; reference range, 6.50-1.50 μg/L) in 3 dogs (13.6%). Serum cobalamin concentrations were decreased (median, 197.6 ng/L; reference range, 250-730 ng/L) in 3 dogs (13.6%). Abdominal ultrasonography identified GI abnormalities in 9 of 23 dogs (39.1%). Five dogs showed signs of gastroenteropathy, characterized by thickening and prominence of the muscularis and submucosal layers of the stomach, along with increased mucosal echogenicity and intramucosal hyperechoic foci in the small intestine. Two dogs had gastropathy characterized by gastric wall thickening and altered layering caused by submucosal prominence. Two dogs exhibited diffuse enteropathy, with intestinal wall thickening and hyperechoic foci within the mucosal layer.

### AGA IgG and TG-2 IgA results at T0

The AGA IgG was positive (median, 1.07) in 12 dogs (52.2%), equivocal (median, 0.65) in 2 dogs (8.7%), and negative (median, 0.33) in 9 dogs (39.1%). The TG-2 IgA was positive (median, 1.20) in 6 dogs (26.1%), equivocal (median, 0.65) in 6 dogs (26.1%), and negative (median, 0.32) in 11 dogs (47.8%). Median AGA IgG titer was 0.86 (range, 0.22-4.18), whereas the median TG-2 IgA titer was 0.61 (range, 0.24-1.89; Table 3).

In total, 14 dogs (60.8%) tested positive for AGA IgG or TG-2 IgA. Four dogs (17.4%) tested positive for both AGA IgG and TG-2 IgA, whereas 1 dog (4.3%) was equivocal for both, and 6 dogs (26.1%) were negative for both.

No significant correlation was found between the number of episodes and either TG-2 IgA or AGA IgG titers at T0 (*P* = .82 and *P* = .89, respectively).

**Table 3:** PD episodes and AGA IgG and TG-2 IgA antibodies evolution in our study population at the 3 observational times.

**Variable**	**T0**	**T1**	**T2**
**PD episodes, median (range)**	6 (2–60)	2 (0–9)	1 (0–10)
**Clinical response, *n* (%):**			
- Excellent	–	7 (30.5%)	10 (43.5%)
- Good	–	12 (52.2%)	10 (43.5%)
- Fairly good	–	2 (8.7%)	1 (4.4%)
- Poor	–	2 (8.7%)	2 (8.7%)
**AGA IgG, median (range)**	0.86 (0.22–4.18)	0.92 (0.2–2.04)	0.75 (0.25–1.90)
-Positive, *n* (%), median	12 (52.2%), 1.07	14 (60.9%), 1.22	10 (43.5%), 1.14
-Questionable, *n* (%), value	2 (8.7%), 0.65	–	2 (8.7%), 0.73
-Negative, *n* (%), median	9 (39.1%), 0.33	9 (39.1%), 0.48	11 (47.8%), 0.42
**TG-2 IgA, median (range)**	0.61 (0.24–1.89)	0.57 (0.18–4.67)	0.63 (0.21–3.18)
-Positive, *n* (%), median	6 (26.1%), 1.20	6 (26.1%), 1.32	8 (34.8%), 1.33
-Questionable, *n* (%), median	6 (26.1%), 0.65	5 (21.7%), 0.68	4 (17.4%), 0.70
-Negative, *n* (%), median	11 (47.8%), 0.32	12 (52.2%), 0.35	11 (47.8%), 0.38

Abbreviations: AGA IgG, anti-gliadin peptide immunoglobulin G; PD, paroxysmal dyskinesia; TG-2 IgA, transglutaminase-2 immunoglobulin A.

“Positive,” “Questionable,” and “Negative” results were classified according to laboratory reference values.

Clinical response: Excellent = complete resolution; Good = ≥50% reduction; Modest = < 50% reduction; and Poor = no response.

Double positive = positive for both AGA IgG and TG-2 IgA at T0. Statistical significance threshold set at *P* < .05.

### Evaluation at T1

At T1, all dogs underwent physical and neurologic examination, which identified no abnormalities. The median number of PD episodes decreased to 2 (range, 0-9) and the median number of PD episodes per dog per month was 0.7 (range, 0-3). The dietary response at T1 was classified as excellent in 7 dogs (30.5%), good in 12 dogs (52.2%), fairly good in 2 dogs (8.7%), and poor in 2 dogs (8.7%; Table 3). The AGA IgG and TG-2 IgA titers at T1 are reported in Table 3, together with the distribution of dogs across positive, equivocal, and negative categories, and the median titers for each.

A total of 18 dogs (78.2%) tested positive for AGA IgG or TG-2 IgA. Two dogs (8.7%) tested positive for both. Four dogs (17.4%) tested negative for both, and none had both AGA IgG and TG-2 IgA classified as equivocal.

No significant correlation was found between the number of episodes and either AGA IgG or TG-2 IgA titers at T1 (*P* = .13 and *P* = .79, respectively; Table 3).

The response to GFHD is summarized in Table 4, together with the median (range) AGA IgG and TG-2 IgA titers at T1.

**Table 4:** Response to gluten-free hydrolyzed diet and corresponding median (range) AGA IgG and TG-2 IgA results at T1 and T2.

**Response to gluten-free hydrolyzed diet**	**No. of dogs (%) at T1**	**AGA IgG at T1 median (range)**	**TG-2 IgA at T1 median (range)**	**No. of dogs (%) at T2**	**AGA IgG at T2 median (range)**	**TG-2 IgA at T2 median (range)**
Excellent	7 (30.5%)	0.90 (0.23–4.18)	0.66 (0.40–1.25)	10 (43.8%)	0.48 (0.25–2.35)	0.43 (0.21–1.38)
Good	12 (52.2%)	0.85 (0.22–3.04)	0.62 (0.38–1.89)	10 (43.8%)	0.75 (0.29–3.62)	0.83 (0.27–3.18)
Fairly good	2 (8.7%)	0.32 (0.31–0.32)	0.48 (0.44–0.51)	1 (4.4%)	1.84	3.03
Poor	2 (8.7%)	0.71 (0.50–0.93)	0.35 (0.24–0.46)	2 (8.7%)	0.92 (0.37–1.90)	0.60 (0.29–0.71)

Abbreviations: AGA IgG, anti-gliadin peptide immunoglobulin G; TG-2 IgA, transglutaminase-2 immunoglobulin A.

### Evaluation at T2

At T2, physical and neurologic examinations were unremarkable, and all dogs accepted the GFHD. The median number of PD episodes was 1 (range, 0-10) and dogs experienced a median of 0.3 episodes per month (range, 0-3.3). Dietary response was classified as excellent in 10 dogs (43.8%), good in 10 dogs (43.8%), fairly good in 1 dog (4.4%), and poor in 2 dogs (8.7%) as reported in Table 3. Table 3 also presents AGA IgG and TG-2 IgA antibody titers at T2, along with the number of dogs classified as positive, equivocal, or negative, and the corresponding median titers for each category.

Twelve dogs (52.2%) tested positive for either AGA IgG or TG-2 IgA. Five dogs (21.7%) tested positive for both. Seven dogs (30.4%) tested negative for both, whereas 2 (8.7%) had AGA IgG and TG-2 IgA results that were classified as equivocal.

No significant correlation was found between the number of episodes and either TG-2 IgA or AGA IgG titers at T1 and T2 (*P* = .34 and *P* = .81, respectively; Table 3; Supplementary Material S3: number of PD episodes and corresponding AGA IgG and TG-2 IgA titers at T0, T1, and T2 for each single dog).

Table 4 presents the response to GFHD as well as the median (range) AGA IgG and TG-2 IgA titers at T2. When T1-T2 interval was considered and compared with the 6-month period before the GFHD trial interval, 10 dogs (43.5%) had an excellent response, 10 dogs (43.5%) had a good response, 1 dog (4.3%) had a fairly good response, and 2 dogs (8.7%) had a poor response (Table 3).

No significant difference was found in the number of episodes per dog per month between the T0-T1 and T1-T2 periods (*P* = .14).

At T2, 1 dog (4.3%) had more than one GI sign, 5 dogs (21.7%) had a single GI sign, and 17 (74.0%) had no GI signs. The specific signs sporadically observed were vomiting (4 dogs), diarrhea (2 dogs), sialorrhea (1 dog), and nausea (1 dog). No borborygmi or indirect signs of abdominal pain were reported at this time point.

### Comparison between T0 and T2

Overall, the median number of PD episodes was 2 (range, 0-10), and the median number of PD episodes per dog per month at 6 months after the introduction of a GFHD was 0.5 (range, 0-1.7). A significant decrease in both the overall number of PD episodes and the number of episodes per month was observed when comparing the 6 months preceding the administration of the GFHD to the 6-month period under dietary intervention (*P* = .0003 and *P* = .001, respectively).

Five dogs (21.7%) had an excellent response to the GFHD with no further episodes, 12 dogs (52.2%) showed a good response, with a decrease of more than 50% in frequency of the episodes, and 6 dogs (26.1%) had a poor response. Five dogs (21.7%) had a good response at T1, and no additional episodes were recorded between T1 and T2. Overall, 17 dogs (73.9%) were classified as having an excellent or good response, defined as a ≥ 50% decrease in PD episode frequency.

Regarding the AGA IgG and TG-2 IgA titers, statistical analysis did not identify a significant decrease between T0 and T2 (*P* = .32 for AGA IgG and *P* = .41 for TG-2 IgA; Table 3: evolution of the clinical response and AGA IgG and TG-2 IgA titers throughout the study period). Statistical analysis identified a significant decrease in vomiting and diarrhea when comparing the 6 months before and after introduction of the GFHD (*P* = .01). However, no significant differences were identified for sialorrhea, nausea, borborygmi or abdominal pain (*P* > .05 for all comparisons).

## Discussion

The main goal of our prospective study was to evaluate the effects of a GFHD on the number of PD episodes in dogs of different breeds over a 6-month period. The results indicate that this dietary treatment is associated with a significant decrease in the number of PD episodes (*P* = .0003). Six months after the introduction of the GFHD, 17 dogs (73.9%) were classified as excellent or good responders, having experienced a decrease in the frequency of PD episodes. Five dogs (21.7%) experienced a complete disappearance of PD episodes, and 12 (52.2%) had a decrease in PD frequency of at least 50%. When the number of PD episodes per month was evaluated, a significant decrease was observed between the 6 months before and the 6 months after introduction of the GFHD (*P* = .001). However, no further decrease in the number of PD episodes per month was observed when comparing T0-T1 with T1-T2 (*P* = .14), suggesting that the response to GFHD occurred mainly during the first 3 months of the dietary trial.

The mechanisms underlying gluten sensitivity are largely unknown in veterinary medicine. However, this condition is well-established in humans as a systemic autoimmune disorder involving an abnormal immune response that presents with a variety of clinical signs, including those affecting the GI tract, skin, and nervous system.^19^

Our study supports previous observations suggesting that gluten-free diets positively impact the frequency of PD episodes.^4–6^^,^^10–12^ However, our results provide more robust evidence because of the study’s prospective design and rigorous inclusion criteria, which included a diagnostic threshold comparable to tier 2 for idiopathic epilepsy, absence of any other pharmacological treatment, and analytical examination of video recordings of the episodes.

Several breeds, including BTs,^4–6^ Maltese dogs,^11^ Shetland Sheepdogs,^10^ and Welsh Terriers,^12^ have been reported in the veterinary literature to have decreased PD episodes after the introduction of a gluten-free diet. The use of gluten-free diets to treat PD in dogs was first reported in 2015 in a prospective study of a small number of BTs.^6^ In this study, all 6 dogs showed a positive response to the gluten-free diet, with absence or decrease of the PD episodes at the 9-month follow-up. Information on the possible effects of dietary changes for the same breed is available in a case report publication^5^ and a retrospective case series based on responses to an online questionnaire.^4^ In the latter study, approximately half of the dogs were reported to have vomiting and diarrhea. Most owners reported changing their BT’s diet after suspecting an association with PD. They opted for hypoallergenic or single-protein and single-carbohydrate diets. This approach resulted in a reported decrease in PD episodes in approximately 50% of the dogs.^4^ In recent years, a few retrospective studies have investigated the effects of gluten-free diets on dogs of other breeds affected by PD.^10–12^^,^^14^ However, these studies often included nonstandardized treatment protocols with gluten-free diets, either alone or in combination with various medications, including antiepileptic drugs, acetazolamide, fluoxetine, muscle relaxants, nonsteroidal anti-inflammatory drugs, and corticosteroids. The study on Shetland Sheepdogs focused on genetic investigations and reported a decrease in PD episodes in 4 dogs after introducing different gluten-free diets.^10^ Another study reported that 6 of 7 Maltese dogs that received a gluten-free diet became free of PD episodes.^11^ In a study on Welsh Terriers,^3^ dogs that were directly observed by the authors were placed on a gluten-free diet, without any change in PD episode frequency.^12^ The study also evaluated owner responses to an online questionnaire and, in some cases, a change in the diet was reported. The authors concluded that “due to the wide variety of nonstandardized interventions provided it was not possible to meaningfully evaluate response to treatment.”^12^

The second goal of our study was to evaluate the changes in AGA IgG and TG-2 IgA titers in the dog population. At the time of admission, AGA IgG was positive in 52.2% and TG-2 IgA was positive in 26.1% of the dogs. When positivity to at least one antibody was considered, 14 dogs (60.8%) had result exceeding 0.8% and 26.1% of the dogs were negative for both antibodies. A recent study regarding PGSD in various breeds found slightly different percentages: 45.16% of dogs were positive for either AGA IgG or TG-2 IgA and 32.26% were negative.^14^

In BTs, reports on positivity to AGA IgG or TG-2 IgA or both show higher percentages. A previous prospective study on 128 BTs, found that AGA IgG and TG-2 IgA antibodies were specific but not sensitive biomarkers for PGSD.^7^ In that study, 45 dogs with suspected PD episodes were compared with 3 groups of dogs with different conditions. The authors demonstrated that 84% (38/45) of dogs with PD had increased AGA IgG titers and 91% (41/45) had increased TG-2 IgA titers. These results were higher than those of the other groups. In the prospective case series, 5 of 6 dogs were positive for AGA IgG and 6 of 6 dogs were positive for TG-2 IgA.^6^ The information is still too limited to draw conclusions on the reason for different percentages of positivity between BTs and other breeds. In our study, AGA IgG and TG-2 IgA titers did not decrease after the introduction of the GFHD, and no significant association was found between AGA IgG and TG-2 IgA results at T0, T1, and T2 and the number of PD episodes at the 3 observation times. These findings are different from those reported in the prospective case series on BTs, where after the introduction of a gluten-free diet, the antibody titers decreased in all dogs.^6^ Our results do not support the hypothesis that AGA IgG and TG-2 IgA titers could be of value in monitoring PD. The longer follow-up period in the previous study might partly explain this discrepancy.^6^ In addition, in our cohort, AGA IgG and TG-2 IgA did not consistently differ between responders and nonresponders to the GFHD. In fact, dogs with excellent or good clinical responses had antibody results that were classified as positive, equivocal, or negative.

This lack of a clear relationship suggests that serum AGA IgG and TG-2 IgA titers, at least in this population, might not be reliable predictors of dietary response. The lack of correlation between antibody titers and clinical response also might reflect the limited availability of serological markers in dogs and the possibility that antigens other than gluten contribute to the underlying disease process. Also, small sample size and uneven distribution across subgroups limit the strength of these conclusions.

In human medicine, serologic biomarkers are commonly employed to support the diagnosis of gluten sensitivity. These biomarkers include AGA and TG-2 antibodies.^20^ Although TG-2 antibodies are specific to gluten-sensitive enteropathy, they are only detected in approximately one-third of patients presenting with neurologic manifestations such as gluten ataxia.^19^ To address this diagnostic limitation, researchers have investigated antibodies against other transglutaminase isoenzymes (eg, TG-3) linked to dermatitis herpetiformis,^21^ and anti-TG-6 antibodies, predominantly found in individuals with neurologic symptoms.^22^ The latter 2 antibody assays are not currently available in veterinary medicine, and their potential role in dogs with PD is unknown. As in human medicine, evaluating other gluten antibodies also might be a useful PGSD diagnostic tool in veterinary medicine. Neurologic signs, such as cerebellar ataxia, myoclonus, and chorea, have been observed in 22.5%-51% of human patients with celiac disease.^23^^,^^24^ Gastrointestinal signs are present in only 10% of cases of celiac disease in humans with neurologic manifestations.^25^ Although the association between PGSD and multisystem manifestations, including GI signs, remains unproven in veterinary medicine, previous studies have shown that some dogs with PD experienced vomiting and chronic diarrhea as non-neurologic signs. Gastrointestinal signs have been reported in BTs with prevalence rates of 48% and 24%^7^ as well as in Welsh terriers (34.1%).^12^ However, these signs have not been thoroughly evaluated.

In our study, we confirmed GI dysfunction on admission in 65% of dogs (15 of 23 dogs; 10 of which had multiple GI signs) based on the presence of one or more GI signs and ultrasonographic findings. Of the 10 affected dogs, 9 (39.1%) had mild GI abnormalities, 5 of which were consistent with mild chronic gastroenteropathy with GI signs found in 7 out of 9 dogs. Overall, decreased serum folate and cobalamin concentrations in 3 dogs suggested the presence of malabsorption associated with chronic enteropathy. All 3 dogs showed GI signs (1 dog with diarrhea, 1 with vomiting and diarrhea, and 1 with diarrhea only). Six months after introducing the GFHD, this percentage decreased to 26% (6 of 23 dogs; 1 of which had multiple GI signs). The most frequently reported GI signs were vomiting and diarrhea, which decreased significantly (*P* = .01) 6 months after introducing the GFHD. This significant decrease suggests that the GFHD might benefit dogs with PGSD and GI signs. The effectiveness of soy-based hydrolyzed diets in dogs with chronic enteropathy is supported by good evidence. These diets have improved clinical signs, decreased clinical activity scores, and led to weight gain,^26–30^ as well as modulating the microbiome^26^ and improving histopathologic duodenal lesions.^28^ Such clinical benefits have been observed from as early as 2 weeks after diet initiation^26^^,^^27^ and response has been maintained for up to 3 years.^27^ However, this finding does not clarify the potential relationship between GI dysfunction and PGSD.

In human medicine, interactions along the brain–gut axis, modulated by the gut microbiota through immunological, neuroendocrine, and neural mechanism, are thought to represent an important pathogenic factor for hyperkinetic movement disorders in patients with GI signs.^19^^,^^31^^,^^32^ In contrast, in veterinary medicine, it remains unclear whether gastroenteropathies and the microbiota contribute to the pathogenesis of PGSD. Given that GFHD can modulate the gut microbiota, an underlying dysbiosis associated with PD cannot be excluded in dogs with GI signs. If so, dietary management might have contributed to the improvement of both GI signs and PD in our study population. Moreover, in this cohort, AGA IgG and TG-2 IgA titers did not appear to reflect the presence of GI signs. The similar distribution of positive, negative, and equivocal results among dogs with and without GI signs at T0, and the failure to observe a decrease in titers of these antibodies after dietary treatment, suggest that additional factors might play a role in the manifestation of PD episodes in dogs with GI signs. Additional investigations into the role of the microbiota are warranted to explore a potential association between GI signs and PD in dogs. Magnetic resonance imaging of the brain did not identify abnormalities in 78.3% (18/23) of the dogs and changes were observed in 21.7% (5/23). The most common change (observed in 3 dogs) was mild to moderate enlargement of the lateral ventricles. A similar change was observed in 3 dogs in a study of Welsh Terriers with PD.^12^ The observed changes might not have played a role in the development of PD because the MRI findings were considered incidental and not correlated with PD episodes. In the veterinary literature, a single report describes a dog diagnosed with meningoencephalitis of unknown etiology and multifocal MRI lesions associated with paroxysmal non-kinesigenic dyskinesia episodes.^1^ An MRI of the brain is a necessary step in the diagnostic protocol for episodic disorders such as idiopathic epilepsy and PD. However, when approaching these disorders, it is important to understand that the primary purpose of MRI is to rule out structural abnormalities. As stated, “The diagnostic yield of advanced neuroimaging techniques in dogs with video footage and historical data supporting PD without neurological deficits is low.”^1^

The limitations of our study include a relatively small sample size, resulting from strict inclusion criteria, and the heterogeneous breed composition, which might include biologically distinct forms of PD. In addition, the absence of an untreated control group prevents discrimination between the effect of the GFHD and spontaneous disease fluctuation, regression to the mean, seasonal variation, or nonspecific effects of dietary modification. No objective means of verifying strict dietary adherence was available, which might have influenced antibody measurements. Furthermore, only episode frequency, and not severity, was evaluated, because no validated severity scale currently exists for dogs with PD. Finally, a dietary re-challenge with a gluten-containing diet was not performed, and therefore, the necessity of lifelong dietary restriction remains unknown. Basal cortisol concentration was not systematically assessed, and hypoadrenocorticism cannot be completely excluded. Another limitation is that epileptic seizures cannot be completely excluded in all cases despite careful video review and normal interictal neurologic examinations, because some epileptic events might closely resemble PD.^33^ The absence of ictal electroencephalography (EEG) further limits diagnostic certainty. As reported in previous studies,^34^ EEG might be useful for ruling out epileptic seizures, one of the most important differential diagnoses. In veterinary neurology, acquiring ictal or interictal EEG is limited by substantial logistical and technical constraints.^33^ These challenges include the low likelihood of recording spontaneous events during the interictal phase and limited access to specialized equipment.^35^ One particularly important topic for further investigation is the prevalence of GI problems in dogs with PD that respond to a GFHD. Although the relationship between PD episodes and chronic gastroenteropathy remains to be explored, our research suggests that a GFHD might improve the GI signs of both conditions. In conclusion, dogs with PD receiving a GFHD showed a significant decrease in PD episode frequency during the study period. However, given the uncontrolled study design, these findings demonstrate an association rather than a causal effect of the dietary intervention. No correlation was found between PD episode frequency and AGA IgG or TG-2 IgA titers. Because the diet was both gluten-free and hydrolyzed, the respective contribution of gluten exclusion and protein hydrolysis to the observed clinical improvement cannot be determined. To date, published studies have focused mainly on gluten-free dietary interventions, whereas the potential role of a hydrolyzed diet itself in dogs with PD has not been investigated. Future controlled studies comparing gluten-free and hydrolyzed diets are warranted to clarify the role of these dietary components.

## Supplementary Material

Supplementary_material_aalag215

## Off-label antimicrobial declaration

The authors declare no off-label use of antimicrobials.

## Institutional animal care and use committee or other approval declaration

Approved by the University of Bologna Ethics Committee (Prot ID 133080/2023). Informed owner consent was obtained.

## Appendix. Materials and methods

### Case selection

The study was designed as a prospective cohort and multicenter investigation.

Dogs were presented to the Veterinary Teaching Hospital of the University of Bologna; Neurovet RSV; Veterinary Center “Pedrani”; Veterinary Neurological Center “La Fenice”; Diagnostic Veterinary Center “Palermovet”; Veterinary Teaching Hospital of the University of Parma; Veterinary Center “Valdinievole” and Veterinary Center “Arcella” between June 2023 and August 2024. The study protocol was approved by the local ethical committee of the University of Bologna (Masked for review) (Prot ID 133080/2023) and written consent was obtained from owners. To be included in the study, dogs were required to meet all of the following criteria:

- History of at least 2 episodes of suspected PD in the 6 months before the neurologic evaluation. Paroxysmal dyskinesia episodes were confirmed by review of video recordings by European College of Veterinary Neurology (ECVN) diplomates or ECVN residents (Supplementary Material S4: video footage of different PD episodes in dogs from our study population).

- Absence of treatment with antiepileptic drugs, serotonin reuptake inhibitors (eg, fluoxetine), carbonic anhydrase inhibitor diuretics (eg, acetazolamide), or corticosteroids.

- Absence of feeding with a gluten-free diet.

- Normal neurologic examination and absence of behavioral changes (as observed by the owner) between PD episodes.

- Normal magnetic resonance imaging (MRI) of the brain or MRI findings not considered relevant or associated with PD. Dogs without video recording of episodes were excluded from the study.

Because of the need to avoid possible influence on GI condition and PD episodes, dogs treated with drugs for underlying GI disorders (gastroprotectants such as antacids, histamine type-2 receptor antagonists, proton pump inhibitors, or antiemetics or probiotics) also were excluded from the study.

### Study design

The study was conducted including 3 observation time points (T): T0 (first presentation and start of the GFHD), T1 (90 days after the introduction of the GFHD), and T2 (180 days after the introduction of GFHD).

At T0, the following history-related information was recorded: breed, sex, age, body weight, 9-point BCS, age of onset of the first episode, episode description (Supplementary Material S2: form with items used to evaluate each PD episode).

The presence of GI signs such as nausea, vomiting, diarrhea, borborygmi, and indirect signs of possible abdominal pain (eg, kyphosis, tense abdomen) also was recorded.

Dogs underwent a thorough physical examination, with particular emphasis on neurologic assessment and evaluation of the GI system.

The following laboratory tests were conducted: CBC, biochemical analysis, serum electrolytes, folate, and cobalamin concentrations, and fecal parasitologic examination. Titration of AGA IgG and TG-2 IgA also was performed. All dogs underwent MRI of the brain and an abdominal ultrasonography.

At T0, after the completion of the clinical assessment, laboratory analysis, and abdominal ultrasonography, dogs were switched to an exclusive commercial soy-based GFHD with a 7-day transition period from the habitual diet. Owners were instructed to maintain standardized diaries documenting PD episode frequency, dietary compliance, and accidental dietary deviations. Preenrollment episode frequency was retrospectively estimated based on owner-maintained records, including smartphone videos, calendars, veterinary reports, and written notes when available.

At T1 and T2, the following information was recorded: occurrence of additional PD episodes, presence of the GI signs, and acceptance of the GFHD. Additionally, comprehensive physical and neurologic examinations were performed, with particular emphasis on the GI tract, as well as AGA IgG and TG-2 IgA titers (Supplementary Material S1: timeline showing the 3 observation periods and diagnostic procedures performed).

The effectiveness of the GFHD was evaluated by comparing the number of episodes in the 6 months before and after the dietary trial. Specifically, the period before the diet began was compared with the T0-T1, T1-T2, and T0-T2 periods. The decrease in episodes was classified as excellent, if there was a complete absence of new episodes; good, if the decrease in episodes was ≥ 50%; fairly good, if the decrease was less than 50%; and poor, if the frequency remained unchanged or increased compared with the 6 months before T0. To classify dogs as responders to the GFHD, excellent and good responses were required, meaning a decrease of at least 50% in the number of PD episodes compared with the pretreatment period.

The AGA IgG and TG-2 IgA results were classified as follows: a titer < 0.6 was considered negative, a titer between 0.6 and 0.8 was considered equivocal, and a titer > 0.8 was considered positive.

The AGA IgG and TG-2 IgA results were evaluated by comparing the titers at T0, T1, and T2.

### Analytical methods

Blood test evaluations were carried out at the Veterinary Laboratory of the University of Bologna and in a reference laboratory (MyLav La Vallonea, Milan, Italy).

The CBC was performed using an automated hematology system (ADVIA 2120, Siemens Healthcare Diagnostics, Tarrytown, NY). Biochemistry was performed using an automated analyzer (OLYMPUS AU480, Olympus/Beckman Coulter, Brea, CA).

Serum cortisol, folate and cobalamin concentrations were measured using chemiluminescent enzyme immunoassay (Immulite 2000, Siemens Healthcare).

Serum samples (with a minimum volume of 0.5 mL) for AGA IgG and TG-2 IgA titration were stored frozen and protected from light until shipment to a commercial laboratory within 2 days and analyzed using a standardized ELISA test (Laboklin GmbH & Co.KG, Labor für Klinische Diagnostik, Adresse: Steubenstraße 4, 97688 Bad Kissingen, Germany).

### Statistical analysis

All data were recorded in a Microsoft Excel for MAC spreadsheet (v. 16.58, Albuquerque, NM) before being imported into statistical analysis software (GraphPad Software Inc., La Jolla, CA). Continuous variables were assessed for normality using the Shapiro–Wilk test. Data related to history and clinical findings were presented as numerical values (percentages) or as the mean or median (ranges), depending on whether the analyzed variables were categorical or continuous.

The Friedman test was used to analyze variations in gluten antibody titer across the 3 observation time points, evaluating AGA IgG and TG-2 IgA separately. The difference in the number of episodes across the 3 observation time points was assessed using the Friedman test. The correlation between AGA IgG and TG-2 IgA titers and episode frequency at the 3 observation time points was evaluated using Spearman’s correlation. The Wilcoxon signed-rank test was used to compare the median number of episodes per month between the 6 months before and 6 months after the introduction of the diet, as well as between the T0-T1 and T1-T2 intervals.

The proportion of dogs with GI signs at T0 and T2 was compared using McNemar’s test. Given the exploratory nature of the study and the limited sample size, correction for multiple comparisons was not applied. Statistical significance was set at *P* < .05 throughout.

## Abbreviations

AGA IgG: anti-gliadin peptide immunoglobulin G
GI: gastrointestinal
GFHD: gluten-free hydrolyzed diet
PD: paroxysmal dyskinesia
PGSD: paroxysmal gluten-sensitive dyskinesia
TG-2 IgA: transglutaminase-2 immunoglobulin A
