# Supplementary material for: Effects of a gluten-free hydrolyzed diet in dogs with paroxysmal dyskinesia: a prospective cohort study
Source: J Vet Intern Med. 2026 Oct 28;40(5):aalag255. doi: 10.1093/jvimsj/aalag255 (PMC13714782; doi:10.1093/jvimsj/aalag255)
Supplement: Supplementary file 1 [file supplementary_material_aalag215.zip › Supplementary material 1 Study design.docx]

# STUDY DESIGN

## T0

Clinical evaluation (physical and neurological examination), blood exams, abdominal ultrasound, MRI of the brain and antibodies titration

## T1 (day G0)

Clinical evaluation (physical and neurological examination), assessment of clinical response to hydrolyzed gluten-free diet and second antibodies titration

## T2 (day 180 )

Clinical evaluation (physical and neurological examination), assessment of clinical response to hydrolyzed gluten-free diet and third antibodies titration

## T3 (day 270)

Analysis of the data obtained

Start of hydrolyzed gluten-free diet
