# Supplementary material for: Effects of a gluten-free hydrolyzed diet in dogs with paroxysmal dyskinesia: a prospective cohort study
Source: J Vet Intern Med. 2026 Oct 28;40(5):aalag255. doi: 10.1093/jvimsj/aalag255 (PMC13714782; doi:10.1093/jvimsj/aalag255)
Supplement: Supplementary file 1 [file supplementary_material_aalag215.zip › Supplementary material 3 Number of PD Episodes and Corresponding AGA IgG and TG2 IgA Values at T0, T1 and T2.docx]

| **Dog** | **Number of PD episodes** | | | **AGA IgG value** | | | **TG2 IgA value** | | |
| --- | --- | --- | --- | --- | --- | --- | --- | --- | --- |
|  | **T0** | **T1** | **T2** | **T0** | **T1** | **T2** | **T0** | **T1** | **T2** |
| **1** | 4 | 0 | 0 | 0.95 | 0.48 | 0.44 | 0.4 | 0.37 | 0.31 |
| **2** | 3 | 2 | 1 | 0.33 | 0.57 | 0.37 | 0.44 | 0.57 | 0.71 |
| **3** | 10 | 1 | 2 | 0.85 | 0.86 | 0.32 | 0.44 | 0.28 | 0.27 |
| **4** | 20 | 1 | 0 | 0.85 | 0.89 | 1.01 | 0.4 | 0.78 | 0.43 |
| **5** | 20 | 0 | 10 | 0.23 | 0.5 | 0.38 | 0.46 | 1.32 | 0.7 |
| **6** | 3 | 2 | 2 | 0.31 | 0.85 | 0.78 | 0.51 | 0.49 | 0.71 |
| **7** | 5 | 0 | 0 | 0.42 | 0.56 | 0.42 | 0.66 | 0.98 | 0.43 |
| **8** | 3 | 0 | 1 | 1.68 | 1.29 | 0.47 | 1.25 | 0.68 | 1.27 |
| **9** | 5 | 0 | 0 | 0.66 | 0.8 | 0.48 | 0.63 | 0.3 | 0.21 |
| **10** | 8 | 2 | 1 | 0.33 | 0.37 | 0.68 | 1.89 | 2.08 | 3.18 |
| **11** | 2 | 1 | 3 | 0.47 | 0.2 | 0.57 | 0.29 | 0.29 | 0.57 |
| **12** | 10 | 3 | 0 | 0.86 | 1.64 | 0.82 | 1.14 | 0.65 | 0.92 |
| **13** | 3 | 1 | 0 | 0.87 | 1.3 | 1.84 | 0.61 | 4.67 | 3.03 |
| **14** | 6 | 0 | 0 | 4.18 | 0.42 | 0.25 | 0.66 | 0.18 | 0.29 |
| **15** | 4 | 2 | 0 | 3.04 | 1.99 | 2.35 | 1.62 | 0.77 | 0.46 |
| **16** | 4 | 0 | 0 | 1.2 | 0.94 | 1.02 | 1.03 | 0.9 | 1.38 |
| **17** | 12 | 1 | 1 | 2.16 | 2.04 | 3.62 | 0.72 | 0.32 | 1.8 |
| **18** | 60 | 4 | 3 | 1.19 | 0.99 | 0.92 | 0.64 | 0.43 | 0.83 |
| **19** | 11 | 11 | 3 | 0.5 | 1.63 | 1.06 | 0.24 | 0.27 | 0.29 |
| **20** | 6 | 2 | 0 | 0.22 | 0.31 | 0.29 | 1.03 | 1.32 | 0.84 |
| **21** | 9 | 1 | 1 | 0.31 | 0.58 | 0.42 | 0.38 | 0.39 | 0.57 |
| **22** | 6 | 3 | 3 | 0.64 | 1.22 | 1.22 | 0.29 | 0.38 | 0.38 |
| **23** | 3 | 5 | 5 | 0.93 | 0.87 | 1.9 | 0.46 | 0.63 | 0.63 |

Supplementary material 3. Number of PD Episodes and Corresponding AGA IgG and TG2 IgA Values at T0, T1, and T2.

**Abbreviations:** PD = Paroxysmal Dyskinesia; AGA = Anti-Gliadin Antibody; TG2 = Tissue Transglutaminase 2; T0 = observational time point 0 (before introducing gluten-free diet); T1 = observational time point 1 (three months after introducing gluten-free diet); T2 = observational time point 2 (six months after introducing gluten-free diet).
